# Hybrid Al_2_O_3_-CH_3_NH_3_PbI_3_ Perovskites towards Avoiding Toxic Solvents

**DOI:** 10.3390/ma13010243

**Published:** 2020-01-06

**Authors:** Eurig Wyn Jones, Peter James Holliman, Leon Bowen, Arthur Connell, Christopher Kershaw, Diana Elizabeth Meza-Rojas

**Affiliations:** 1 College of Engineering, Swansea University, Bay Campus, Swansea SA1 8EN, UK; p.j.holliman@swansea.ac.uk (P.J.H.); arthur.connell@swansea.ac.uk (A.C.); c.p.kershaw@swansea.ac.uk (C.K.); d.e.mezarojas@swansea.ac.uk (D.E.M.-R.); 2 Physics Department, Durham University, Durham DH1 3LE, UK; leon.bowen@durham.ac.uk

**Keywords:** perovskite, upscaling, lifetime, humidity, coating, stability, non-toxic

## Abstract

We report the synthesis of organometal halide perovskites by milling CH_3_NH_3_I and PbI_2_ directly with an Al_2_O_3_ scaffold to create hybrid Al_2_O_3_-CH_3_NH_3_PbI_3_ perovskites, without the use of organic capping ligands that otherwise limit the growth of the material in the three dimensions. Not only does this improve the ambient stability of perovskites in air (100 min versus 5 min for dimethylformamide (DMF)-processed material), the method also uses much fewer toxic solvents (terpineol versus dimethylformamide). This has been achieved by solid-state reaction of the perovskite precursors to produce larger perovskite nanoparticles. The resulting hybrid perovskite–alumina particles effectively improve the hydrophobicity of the perovskite phase whilst the increased thermal mass of the Al_2_O_3_ increases the thermal stability of the organic cation. Raman data show the incorporation of Al_2_O_3_ shifts the perovskite spectrum, suggesting the formation of a hybrid 3D mesoporous stack. Laser-induced current mapping (LBIC) and superoxide generation measurements, coupled to thermogravimetric analysis, show that these hybrid perovskites demonstrate slightly improved oxygen and thermal stability, whilst ultra-fast X-ray diffraction studies using synchrotron radiation show substantial (20×) increase in humidity stability. Overall, these data show considerably improved ambient stability of the hybrid perovskites compared to the solution-processed material.

## 1. Introduction

The meteoric rise in operating efficiency of perovskite solar cells (PSCs) from ca. 12% in 2012 [1] to 23% [2] has generated enormous interest in the technology. In addition, whilst a 1 year PSC device has been reported [3], issues remain regarding their ambient stability. There is also a need to consider lead-free PSC devices, which has led researchers to substitute Pb with other metals (e.g., Sn) [4] whilst also adding other ions (e.g., Cs, etc. [5]) to improve stability. However, to date, reports which have moved away from Pb-based perovskites have led to an increased likelihood of oxidative and/or hydrolytic degradation leading to oxide, hydroxide or precursor breakdown products [6]. To counteract this, some level of oxidation mitigation has been achieved [7] through pairing Sn with Pb to stabilize the Sn^2+^ in its +II oxidation state [8]. More recently, researchers have produced all-Sn perovskite without using any Pb to stabilize the crystal by using ultra-high-purity starting materials, and a highly controlled, low-humidity atmosphere [6].

**Figure 1:**
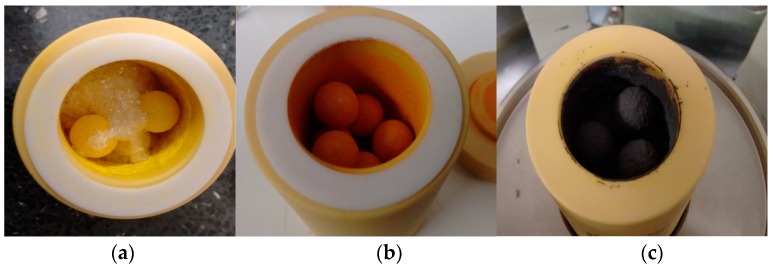
Image of zirconia ball mill vessel during hybrid Al_2_O_3_-CH_3_NH_3_PbI_3_ perovskite synthesis, showing (**a**) yellow PbI_2_ and white CH_3_NH_3_I, (**b**) initial precursor with Al_2_O_3_ and (**c**) black hybrid Al_2_O_3_-CH_3_NH_3_PbI_3_ perovskite.

In order for scale up and to prove the efficacy of PSC technology, low-cost raw materials and processing need to be developed, which leads to stable absorber materials. In this paper, we report all-ambient synthesis and processing of organolead perovskites by pairing the perovskite precursors (CH_3_NH_3_I and PbI_2_) directly with alumina and then mixing the pre-made hybrid Al_2_O_3_-CH_3_NH_3_PbI_3_ with benign terpineol solvent, affording a hybrid metal oxide–perovskite ink (Figure 1). In our initial paper [9], we reported the deposition of a thick layer of this hybrid mesoporous perovskite stack using blade casting. This produced a non-uniform layer with large voids. Despite this, current-voltage (I–V) (Table 1) and photoluminescence (PL) data showed that the oxide–perovskite layer was effective at injecting and transporting electrons. Recent studies [10] have shown that grain boundaries/voids do not seem to generate gap states in organolead perovskites, which considerably relaxes material quality constraints for both material purity and deposition method. 

Following our initial publication, another group [11] reported a similar method, whereby perovskite nanoparticles were suspended, and then sprayed down, followed by a compression step to further compact the layer and increase the contact area between the hole transport layer and the perovskite. This gave device efficiencies over 9% and further demonstrates the potential of processing pre-suspended perovskite nanoparticles. Al_2_O_3_ is also previously reported to be an effective barrier to moisture and has been used as a capping layer to shield the perovskite from humidity [12] whilst its use as a mesoporous scaffold has been shown to improve perovskite device stability [13,14,15].

In terms of perovskite stability, it has been reported that solvent-processed perovskites (dimethylformamide—DMF, dimethylsulfoxide—DMSO, etc.) start to decompose within 2 h and totally decompose within 24 h [16]. Whilst the initial use of Al_2_O_3_ in perovskite cells was as a mesoporous electron transport layer (although this has now more commonly been replaced by a thin “blocking” layer of TiO_2_ to increase the Fermi level (E_F_) [17]) it also has implications for device lifetime by counter-acting the p-type doping of the perovskite degradation species. In practice, the E_F_ increase can be thought of as effectively doping the perovskite further n-type either as a result of high fixed negative charge density of Al_2_O_3_ under-coordinated I^−^ ions, or that the Al_2_O_3_ scaffold passivates or even fills traps at the perovskite interface (similar to the phenomenon exploited in Si photovoltaics (PV) to passivate surface states [18]). 

In this paper, we have studied the synthesis of hybrid Al_2_O_3_-CH_3_NH_3_PbI_3_ perovskites using a solid-state milling process in the absence of any processing solvent. These particles have then been suspended in terpineol to make a hybrid metal oxide–perovskite ink. We have then studied the degradation lifetime and mechanisms (e.g., O_2_, thermal and humidity stability) of this material versus a solvent-processed (dimethylformamide—DMF) CH_3_NH_3_PbI_3_ perovskite. 

## 2. Experimental Procedures 

### 2.1. Hybrid Al_2_O_3_-Perovskite Synthesis

Hybrid Al_2_O_3_-CH_3_NH_3_PbI_3_ perovskite was synthesised as described in ref. [9] by first grinding PbI_2_ (1.0 g) with Al_2_O_3_ nanoparticles (0.4 g, mean particle size 13 nm) in a Fritsch P6 ball mill with a zirconia vessel at 400 rpm; leading to ca. 13% Al_2_O_3_ wt/wt loading. CH_3_NH_3_I (1.679 g) was then added and the samples were reground together for 10 min with relative humidity ≤40%. The hybrid Al_2_O_3_-CH_3_NH_3_PbI_3_ perovskite powder was suspended in α-terpineol (1.5 mL, Aldrich, Gillingham, UK) by mixing for 24 h followed by sonication for 12 h to ensure de-agglomeration. The resulting hybrid perovskite inks were deposited in a fume hood onto TEC-7 (NSG) glass which had been pre-coated with a compact c-TiO_2_-blocking layer using bar coater (RK Print K101 Control Coater). The deposited films were sintered from ambient to 70 °C at 3 °C/min with 10 min dwell, then ramped to 120 °C at 10 °C/min with 40 min dwell before cooling to room temperature.

### 2.2. Synchrotron X-ray Powder Diffraction (XRD) Measurements

**Figure 2:**
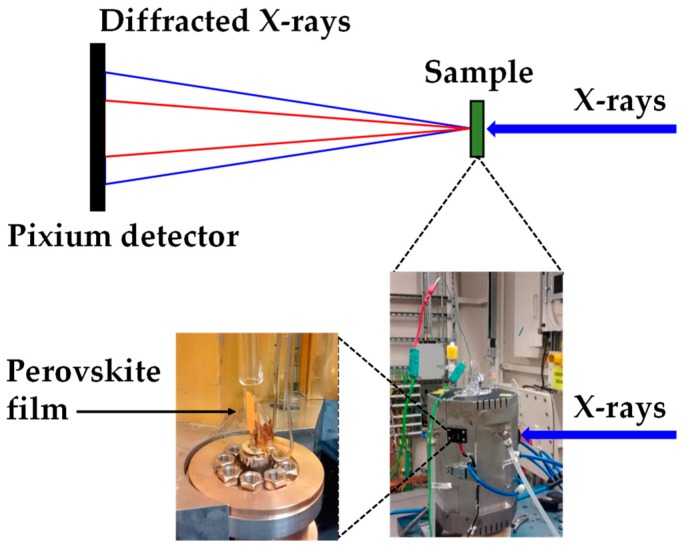
Experimental set up at the Diamond Light Source synchrotron to measure ultrafast X-ray powder diffraction (XRD) showing the Oxford-Diamond In Situ Cell (ODISC) furnace with gas lines with (**left**) the custom quartz, humidity variable reactor and (**right**) ODSIC near infrared furnace.

Ultrafast synchrotron XRD measurements (Figure 2) were taken at Diamond Light source (Experiment No: EE11161.1) using beamline I:12, utilizing a Pixium RF4343 detector. Humidity levels were controlled using a GenRH humidity generator (Surface Measurement Systems, London, UK) and flowing the gas into a custom-made glass reactor placed inside the custom-made Oxford-Diamond In Situ Cell (ODISC) near infrared (NIR) furnace. Temperatures were monitored using a thermocouple. X-ray wavelength: 0.224 Å (55.35 keV, detector distance 2023.49 cm).

### 2.3. Raman Spectroscopy

Scans were made using a Renishaw InVia Confocal Raman Microscope (laser λ = 532 nm) taken at 0.05% laser intensity for 10 s at 50× magnification. PL data were measured using 0.0001% laser intensity) on sintered thin films of bare perovskite; i.e., no hole transport material or surface protection layer was employed.

### 2.4. Thermal Analysis

A TA Instruments Q600 SDT (TA Instruments, Elstree, UK) with dry air and controlled humidity, using a GenRH humidity controller was used to study weight and heat flow at various humidity levels at a 10 °C min^−1^ ramp rate. 

### 2.5. Electron Beam-Induced Current (EBIC) Mapping

**Figure 3:**
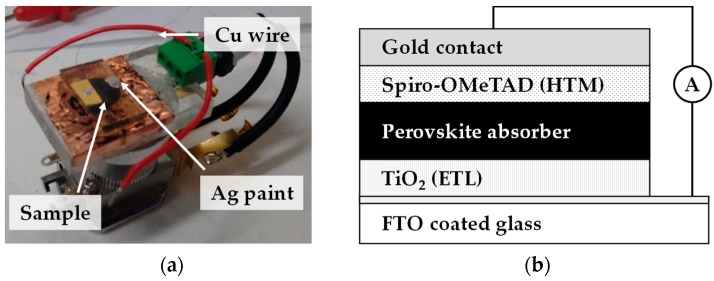
Image showing (**a**) sample holder and (**b**) schematic of the corresponding device configuration for Electron Beam-Induced Current (EBIC) measurements. Connections are made using silver paste and thin copper wire is used to electrically connect the cell to the custom Scanning Electron Microscopy (SEM)-EBIC holder.

EBIC was carried out using a multimode SEM with an EBIC amplifier as shown in Figure 3. Before analysis, ion beam milling was carried out using an FIB milling beam for 30 min to produce as ample cross-section. Samples were connected using silver paint with copper wire electrodes. Sample cross-sections were platinum coated to minimise charging in the electron beam.

### 2.6. Superoxide Evolution

Superoxide evolution measurements were made using the set-up reported previously by Aristidou et al. [19], which allows direct measurement of superoxide generation from a sample. Photoluminescence spectra were recorded using an excitation wavelength of 520 nm and a slit width of 10 nm on a Horiba Jobin-Yvon Fluorolog-3 Spectro fluorimeter (Horiba UK Ltd., Northampton, UK). 

## 3. Results and Discussion

### 3.1. Scanning Electron Microscopy (SEM) and Electron Beam-Induced Current (EBIC) Mapping

**Figure 4:**
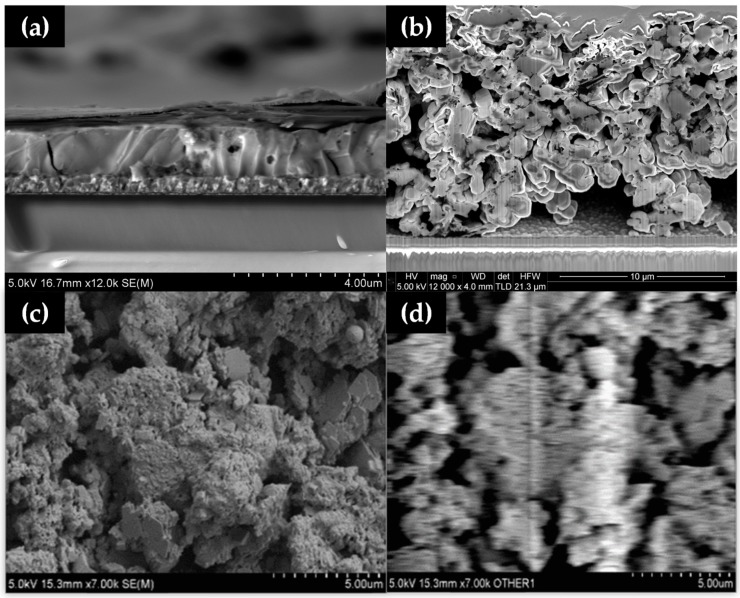
Cross-sectional SEM of (**a**) dimethylformamide (DMF)-processed perovskite and (**b**) a blade cast hybrid Al_2_O_3_-CH_3_NH_3_PbI_3_ perovskite without a compression step showing large voids and (**c**) SEM and (**d**) EBIC images of hybrid Al_2_O_3_-CH_3_NH_3_PbI_3._

Cross-sectional SEM images (Figure 4) of solution-processed perovskite versus hybrid Al_2_O_3_-CH_3_NH_3_PbI_3_ perovskite film show a markedly different morphology, with spin coating producing a compact perovskite film (Figure 4a) with the hybrid perovskite film showing large (2–4 µm) grains and voids (Figure 4b), and a much larger (10–12 µm) film thickness. This suggests that hybrid Al_2_O_3_-CH_3_NH_3_PbI_3_ perovskite films made without a compression step are not well suited to make PV devices but, because this is less relevant here because this study is focussed on material stability.

Electron Beam-Induced Current (EBIC) mapping was carried out on the mesoporous stack (Figure 4c,d). Although it was expected that the work function of the material would shift in the vacuum of the SEM, which did make direct electrical observation difficult, an EBIC signal was observed from the material (shown as the white region in Figure 4d). This was despite observing buried junction behaviour towards the back contact of the device, which showed up as increases in current when the light was turned on inside the chamber. EBIC data showed that the layer thickness and large voids in the layer limited the amount of current collected by the contacts, showing only marginal current activity.

### 3.2. Material Stability to Oxygen

**Figure 5:**
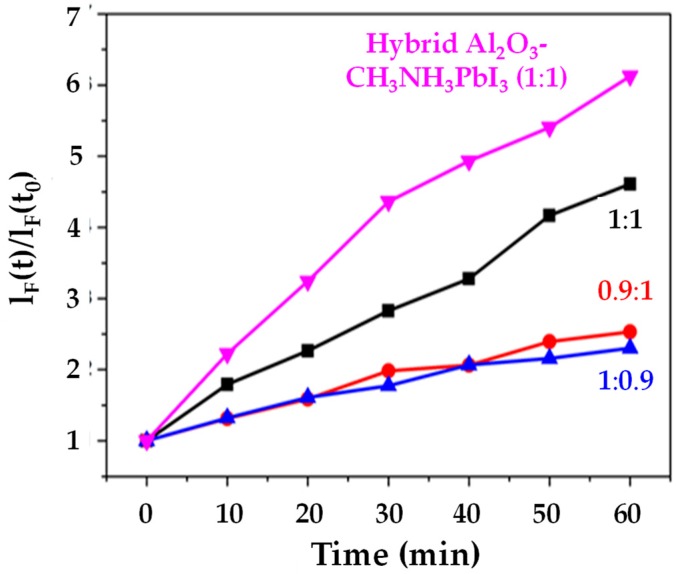
Superoxide production versus time for CH_3_NH_3_PbI_3_ samples with varying PbI_2_:CH_3_NH_3_PbI ratios of 1:1, 0.9:1 1:0.9) and hybrid Al_2_O_3_-perovskite (PbI_2_: CH_3_NH_3_PbI ratio of 1:1).

We have studied light-driven superoxide generation in CH_3_NH_3_PbI_3_ films because this organolead halide perovskite should be the least stable compared to other halide (Cl^−^, Br^−^ or I^−^) or mixed halide systems. This was based on the fact that Aristidou et al. [19] have previously shown that, during exposure of CH_3_NH_3_PbI_3_ films to light and O_2_, the first step of the degradation mechanism involves the transfer of photogenerated CH_3_NH_3_PbI_3_ electrons to molecular O_2_, resulting in the formation of superoxide (O_2_^−^). This superoxide then goes on to react with the methylammonium moiety of the perovskite absorber. Iodide [20] has also been reported to limit the O_2_ and light stability of CH_3_NH_3_Pb(I_1x_Br_x_)_3_ perovskites, with perovskites containing less or no I^−^ being more stable. Therefore, a CH_3_NH_3_PbI_3_ film was used as the first (control) sample. The data (Figure 5) show that the CH_3_NH_3_PbI_3_ film generates less than half the superoxide than the supposedly more stable I-Br hybrid [20]. This is despite the CH_3_NH_3_PbI_3_ films being much thicker (5–8 µm) which means that, in theory, they should generate more superoxide. Figure 5 also shows that when an equimolar ratio of PbI_2_:CH_3_NH_3_I is used, an increase in superoxide is generated as opposed to when the perovskite stack is PbI_2_ or CH_3_NH_3_I deficient. This result was not expected, as Pb or halide deficiencies typically increase degradation, such that excess PbI_2_ or CH_3_NH_3_I is usually used to minimise degradation [21]. Figure 5 also shows that the hybrid Al_2_O_3_-CH_3_NH_3_PbI_3_ film appears to generate more superoxides relative to the other samples. This result was unexpected, as we have previously reported [9] that samples with integrated alumina are inherently more structurally stable. This result indicates that the increased stability of the hybrid Al_2_O_3_-CH_3_NH_3_PbI_3_ perovskite may not arise from less superoxide generation from the ca. 8 µm thick hybrid stack. The concomitant increase in superoxide generation alongside the increased structural stability of the hybrid Al_2_O_3_-CH_3_NH_3_PbI_3_ can be rationalized by looking at recent work by Pont et al. [22], which stipulates that the reason superoxide does not degrade their more stable CH_3_NH_3_PbBr_3_ films is due to an increased thermodynamic stability of the bromide crystal structure, compared to CH_3_NH_3_PbI_3_. Hence, this could suggest increased thermodynamic stability of the hybrid Al_2_O_3_-CH_3_NH_3_PbI_3_ film over the neat CH_3_NH_3_PbI_3_ one. Further support for this arises from studies of the thermal stability of perovskite with differing deposition techniques [23], which confirms subtle differences in the crystal phase depending on the method of deposition used.

### 3.3. Thermal Analysis

**Figure 6:**
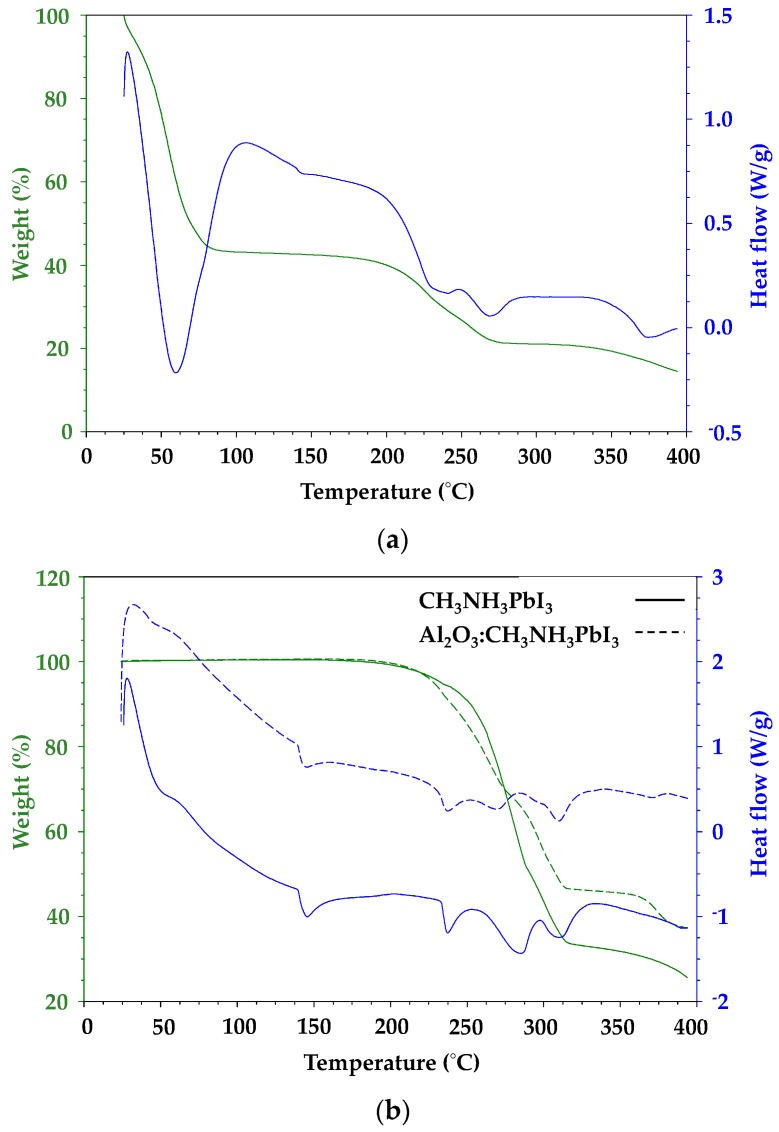
(**a**) Thermal gravimetric analysis (TGA) weight loss data measured in air for dimethylformamide (DMF) solution-processed CH_3_NH_3_PbI_3_ (**b**) DSC data showing heat flow for solid-state synthesised CH_3_NH_3_PbI_3_ (solid line) and hybrid Al_2_O_3_-CH_3_NH_3_PbI_3_ (dash line).

Thermal analysis was carried out on perovskite samples with varying PbI_2_:CH_3_NH_3_I ratios to simulate iodine deficiency or excess. The thermal gravimetric analysis (TGA) data (Figure 6) show a single weight loss event for the organic component along with a sharp endotherm in the DSC data for material sublimation. In contrast, the metal halide gives a broad weight loss in line with thermal degradation with the weight loss slowing as the temperature rises which reflects the formation of decomposition products which degrade more slowly [24]. To avoid any overestimation of sublimation or degradation temperature which might arise from a fast heating rate, a 10 °C min^−1^ ramp rate was used for all samples.

Figure 6 shows that the DMF-processed CH_3_NH_3_PbI_3_ shows an initial weight loss of nearly 60% up to ca. 75 °C, which is ascribed to solvent loss followed by initiation of the organic cation breakdown at ca. 190 °C. By comparison, a sample of CH_3_NH_3_PbI_3_ produced by solvent-free milling of CH_3_NH_3_I and PbI_2_ shows no solvent loss and a slight increase in temperature stability, with initiation of organic cation breakdown shifted to ca. 210 °C along with a further shoulder at ca. 280 °C. Hybrid Al_2_O_3_-CH_3_NH_3_PbI_3_ perovskite shows a very similar profile to this with organic cation breakdown at 210 °C along but with a further stabilisation of the 280 °C feature. Figure 6b shows heat flow signal versus temperature for solvent-free milled CH_3_NH_3_PbI_3_ which has been milled either with or without Al_2_O_3_. Looking first at the endotherm at ca. 45 °C, which is ascribed to the tetragonal to cubic phase transition for CH_3_NH_3_PbI_3_ perovskite [8], the data show a very slightly earlier onset (45 °C versus 48 °C) and lower enthalpy (4.6 J g^−1^ versus 16.1 J g^−1^) for the hybrid Al_2_O_3_-CH_3_NH_3_PbI_3_ perovskite compared to the CH_3_NH_3_PbI_3_ sample. These trends are also observed for the endothermic peaks at ca. 145 °C and 237 °C. These lower heat flow data for the hybrid Al_2_O_3_-CH_3_NH_3_PbI_3_ perovskite samples presumably reflect the fact that these endothermic signals arise from CH_3_NH_3_PbI_3_ and there is less of this perovskite phase in the hybrid Al_2_O_3_-CH_3_NH_3_PbI_3_ material.

### 3.4. Raman Spectroscopy

**Figure 7:**
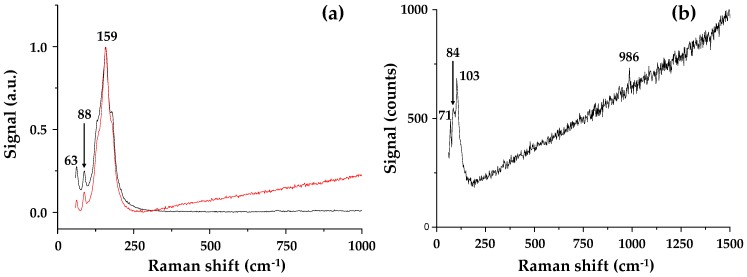
Raman spectra of (**a**) the milled Al_2_O_3_-PbI_2_ sample showing signals from two distinct regions in the sample and (**b**) hybrid Al_2_O_3_-CH_3_NH_3_PbI_3_ perovskite.

The Raman spectrum (Figure 7) of PbI_2_-coated alumina particles show peaks at 63 and 88 cm^−1^ (versus 62 and 94 cm^−1^ for the DMF solution-processed perovskite). These peaks have been assigned to bending and stretching modes of the Pb−I bond (and are diagnostic modes of the inorganic cage) in line with the Raman data interpretation of perovskite species by Quarti et al. [25]. The small peak shifts are in line with previous reports for perovskite adsorption to nanoparticles [26]. The more intense peak at 159 cm^−1^ is near to the attributed vibration for the organic cations (ca. 154 cm^−1^). After addition of methyl ammonium iodide to the PbI_2_-coated alumina nanoparticles, the relative wavenumbers reduce to 71, 84 and 103 cm^−1^, the disappearance of the vibrational mode at 159 cm^−1^ possibly hints at the absence of any excess organic cations, and again, this hints at improved stability. Considering the interaction between the perovskite and Al_2_O_3_ phases, it is interesting to consider that adding the Al_2_O_3_ nanoparticles directly with perovskite precursors and suspending them in the terpineol solvent is similar to what occurs during spin coating. For example, during spin coating, the poor solubility of PbI_2_ in the precursor solution causes PbI_2_ crystals to precipitate first, forming an inorganic framework. This framework then reacts with the organic cation (CH_3_NH_3_I) to form CH_3_NH_3_PbI_3_ crystals. Hence, we believe that perovskite formation in both the spin coating and hybrid Al_2_O_3_-perovskite ink are limited by the rate of PbI_2_ crystallization. However, we believe that it is possible to overcome this limitation in the hybrid Al_2_O_3_-perovskite ink because this is synthesised by ball milling the Al_2_O_3_ with PbI_2_; followed by the addition of the organic cation.

### 3.5. Routine and Ultra-Fast (Synchrotron) X-ray Crystallography

**Figure 8:**
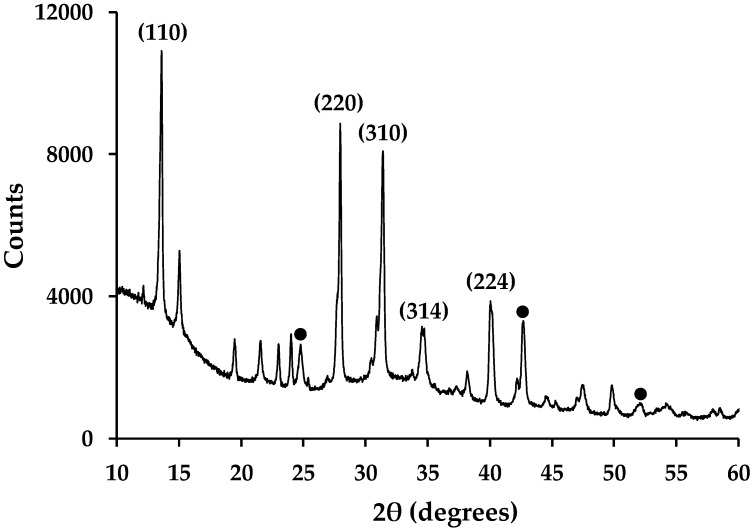
X-ray powder diffraction data of hybrid Al_2_O_3_-CH_3_NH_3_PbI_3_ perovskites showing selected, indexed peaks for CH_3_NH_3_PbI_3_ phase. ● = key peaks from International Centre for Diffraction Data (ICDD) 01-075-1862 XRD pattern for Al_2_O_3._

Routine powder XRD of hybrid Al_2_O_3-_CH_3_NH_3_PbI_3_ perovskite material shows the presence of CH_3_NH_3_PbI_3_ and Al_2_O_3_ (Figure 8) along with small amounts of precursor starting material (PbI_2_ and CH_3_NH_3_I). This is in line with previous reports for organolead perovskites made this way [9,16]. Whilst these data are qualitative rather than quantitative, the peak intensities in Figure 8 do show that there is more perovskite present compared to Al_2_O_3_. This is expected because there is ca. 13% Al_2_O_3_ wt/wt loading in these samples. Whilst this equates to a similar molar ratio of Al_2_O_3_-CH_3_NH_3_PbI_3_ (0.127 moles:0.140 moles), the greater X-ray scattering power of Pb and I will enhance the peak intensities of the perovskite phase. The presence of Pb, C, Al and O within the mesoporous Al_2_O_3_-CH_3_NH_3_PbI_3_ films has also previously been confirmed by cross-sectional EDX mapping of samples produced by FIB-SEM [9].

Ultrafast X-ray powder diffraction data has been used to monitor the humidity susceptibility of the hybrid Al_2_O_3_-perovskites compared to solution-processed materials. Previous work on solvent-processed perovskites has shown that, when exposed to humidity, new diffraction peaks due to the onset of hydrated perovskite (e.g., CH_3_NH_3_PbI_3_·H_2_O) at ~8.6° and 10.5° i.e., d spacings of 1.493 and 1.224 nm (110), respectively [27]. This is in line with reports that intercalation of water molecules into the crystal structure induces rearrangement which separates the [PbI_6_]^4−^ octahedra from a three-dimensional (3D) network of octahedra to one-dimensional (1D) double chains for the monohydrated CH_3_NH_3_PbI_3_·H_2_O and finally into a zero-dimensional (0D) framework of isolated octahedra in the dihydrate (CH_3_NH_3_PbI_3_·2H_2_O). In this context, it is also known that solution-processed perovskites do retain residual processing solvent within the crystal lattice [28]. This residual solvent is likely to exchange with water molecules during humidity exposure and increase the rate of degradation to the hydrated perovskite phases.

**Figure 9:**
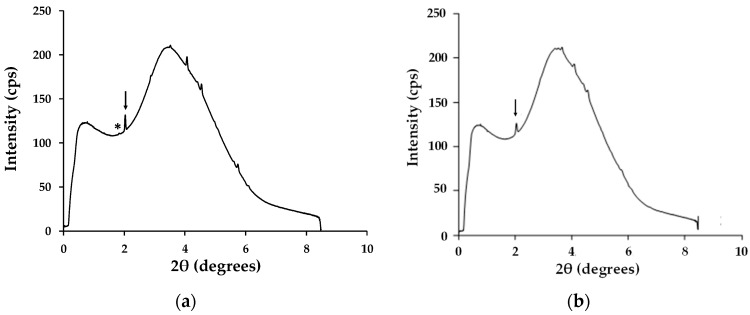
Time-resolved synchrotron X-ray scans processed using Fit2D software for (**a**) DMF solution-processed CH_3_NH_3_PbI_3_ after 5 min and (**b**) hybrid Al_2_O_3-_CH_3_NH_3_PbI_3_ after 60 min. Arrow = CH_3_NH_3_PbI_3_ and * = PbI_2._

**Figure A1:**
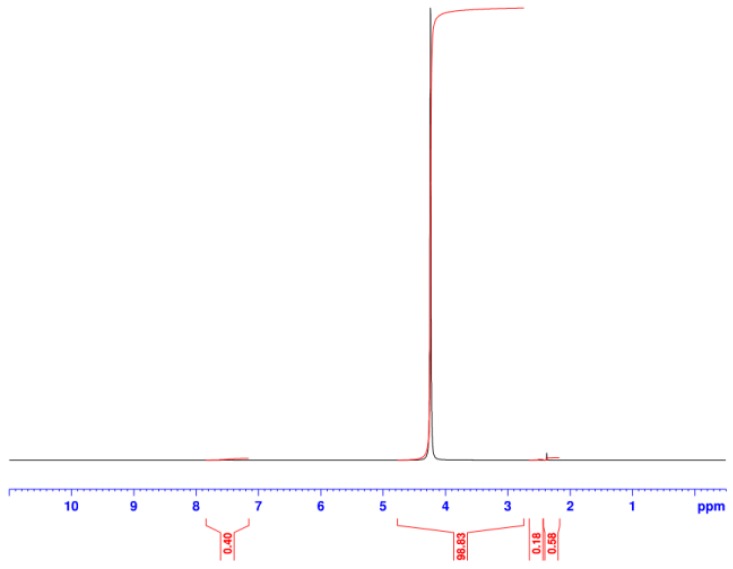
NMR of sintered Al_2_O_3_-CH_3_NH_3_PbI_3_ showing no peak for terpineol (suspension medium) or any residual solvent.

The ultra-fast XRD data (Figure 9) support these assertions by showing show that, for DMF-processed CH_3_NH_3_PbI_3_, that the main peak for the PbI_2_ degradation product can be seen after only 5 min exposure to high humidity. However, with the hybrid Al_2_O_3_-CH_3_NH_3_PbI_3_, the PbI_2_ degradation peak is not observed for over 100 min of high humidity exposure. The key difference here is that, because the hybrid Al_2_O_3_-CH_3_NH_3_PbI_3_ is made by solid-state reaction, there is no residual solvent within the lattice. Instead, the pre-formed hybrid perovskite is suspended in terpineol for deposition and then sintered. So, in this case, because no solvent is present as solvent of crystallisation the sintering process evaporates all the terpineol solvent. This has been studied by blade casting and sintering a terpineol-based hybrid perovskite suspension and then dissolving off the deposit for nuclear magnetic resonance (NMR) analysis (Appendix A, Figure A1). The data show no residual terpineol is present within the sintered hybrid perovskite. Hence, there is no residual solvent in the hybrid perovskite lattice for the water molecules to exchange with and so the rate of humidity-based degradation is significantly reduced by some 20× (i.e., 100 min versus 5 min).

### 3.6. Device Stability Discussion

To explain the enhanced stability of the hybrid material, we propose enhanced sorption of the organic cation (CH_3_NH_3_^+^) to the Al_2_O_3_ nanoparticulate scaffold, which improves metal oxide–perovskite interfacial contact. Related adsorption properties of Al_2_O_3_ have been used extensively in the field of catalytic converters, where heat is used to displace one of the surface-bound oxygen atoms on the Al_2_O_3_ support, leaving it vacant for further sorption [29] and oxidation/reduction [30,31]. It has further been shown that these surface states can be passivated by insertion of a complementary weak Lewis base [32] such as the CH_3_NH_3_^+^ cation used in these materials which would be expected to lower O_2_ or humidity susceptibility by limiting O_2_ or H_2_O ingress to the vulnerable perovskite phase. Related approaches to improve stability have included the use of [Li+@C60] bis(trifluoromethanesulfonyl)imide (TFSI^-^)-doped N2,N2,N2′,N2′,N7,N7,N7′,N7′-octakis(4-methoxyphenyl)-9,9′-spirobi[9H-fluorene]-2,2′,7,7′-tetramine (spiro-OMeTAD) [33] or spiro-OMeTAD-infiltrated carbon nanotubes [34] as the hole transporting layer to increase hydrophobicity. It is also known that transitional aluminas (for example, γ-Al_2_O_3_) have an appreciable Lewis acidity, and that coordinatively unsaturated aluminium ions play a role of Lewis acid sites on the alumina surface [35]. Furthermore, the surface charge of Al_2_O_3_ nanoparticles is dependent on their environment being positive at low pH values, zero at pH 8 and negative at higher pH values [36]. In the context of perovskite formation on a charged Al_2_O_3_ surface, this is likely to define which ions adsorb first during the nucleation stage (I^−^ to a positively charged surface or the cations to a negatively charged surface).

## 4. Conclusions

We have shown that solid-state preparation of CH_3_NH_3_PbI_3_ perovskite directly onto Al_2_O_3_ in the absence of any solvent improves the stability of the resultant hybrid material in the presence of high humidity (80% RH) by 20× (i.e., 100 min versus 5 min). Whilst this is clearly a very substantial improvement, it should also be noted that this is for the most moisture-sensitive organolead halide perovskite studied under constant levels of high humidity. Thus, we would anticipate further enhancements in lifetime for other more stable perovskite phases, e.g., the partial substitution of non-lead ions such as Cl^−^ or Br^−^ for iodide or CH_3_NH_3_^+^ and/or mixed cation (formadinium or caesium) perovskites [37]. Investigations into the reason for the stability enhancement from superoxide generation, Raman spectroscopy and thermogravimetric measurements show subtle differences between the solvent-processed CH_3_NH_3_PbI_3_ and the hybrid Al_2_O_3_-CH_3_NH_3_PbI_3_. However, the clearest difference between these materials is the residual solvent. For example, the DMF-processed perovskite material is known to contain residual solvent, whilst our data suggest that no terpineol is present in the hybrid Al_2_O_3_-CH_3_NH_3_PbI_3._ This absence of solvent appears to significantly retard moisture ingress into the perovskite phase, leading to substantial lifetime enhancement. In addition to this benefit, the production of terpineol-based hybrid Al_2_O_3_-CH_3_NH_3_PbI_3_ inks, combined with the range of compatible roll-to-roll printing technology options available (e.g., screen printing, slot die coating), could allow for a highly scalable, greener perovskite deposition using much lower toxicity solvents [38]. This also opens the possibility to manufacture perovskite solar cells under ambient conditions.

## Figures and Tables

**Table 1:** Current–voltage (I–V) data of Al_2_O_3_-CH_3_NH_3_PbI_3_ perovskite ink devices or solution-processed CH_3_NH_3_PbI_3._

Device	η/%	V_oc_/V	J_sc_/mA cm^−2^	FF	Ref.
Al_2_O_3_-CH_3_NH_3_PbI_3_					
As made	1.1	0.96	2.44	0.45	[9]
After 24 h	1.5	0.98	2.81	0.55	
Aged for 1 month	0.1	0.65	0.51	0.26	
As made (compressed)	9.1	0.92	18.62	0.53	[11]
Solution processed					
As made	5.2	0.83	12.23	0.51	[9]
Aged for 1 month	0.0	N.R.	N.R.	N.R.
